# Interprofessional practices of physiotherapists working with adults with low back pain in Québec’s private sector: results of a qualitative study

**DOI:** 10.1186/1471-2474-15-160

**Published:** 2014-05-17

**Authors:** Kadija Perreault, Clermont E Dionne, Michel Rossignol, Diane Morin

**Affiliations:** 1 Centre for Interdisciplinary Research in Rehabilitation and Social Integration, Institut de réadaptation en déficience physique de Québec, Québec City, Canada; 2 Université Laval, Québec City, Canada; 3 Axe Santé des populations et pratiques optimales en santé, CHU de Québec Research Center, Québec City, Canada; 4 Institut national d’excellence en santé et en services sociaux, Montréal, Canada; 5 Department of Epidemiology, Biostatistics and Occupational Health, McGill University, Montréal, Canada; 6 Institut universitaire de formation et de recherche en soins, Université de Lausanne, Lausanne, Switzerland

**Keywords:** Interprofessional collaboration, Physiotherapy, Physical therapy, Low back pain, Private sector, Qualitative research

## Abstract

**Background:**

Collaboration and interprofessional practices are highly valued in health systems, because they are thought to improve outcomes of care for persons with complex health problems, such as low back pain. Physiotherapists, like all health providers, are encouraged to take part in interprofessional practices. However, little is known about these practices, especially for private sector physiotherapists. This study aimed to: 1) explore how physiotherapists working in the private sector with adults with low back pain describe their interprofessional practices, 2) identify factors that influence their interprofessional practices, and 3) identify their perceived effects.

**Methods:**

Participants were 13 physiotherapists, 10 women/3 men, having between 3 and 21 years of professional experience. For this descriptive qualitative study, we used face-to-face semi-structured interviews and conducted content analysis encompassing data coding and thematic regrouping.

**Results:**

Physiotherapists described interprofessional practices heterogeneously, including numerous processes such as sharing information and referring. Factors that influenced physiotherapists’ interprofessional practices were related to patients, providers, organizations, and wider systems (e.g. professional system). Physiotherapists mostly viewed positive effects of interprofessional practices, including elements such as gaining new knowledge as a provider and being valued in one’s own role, as well as improvements in overall treatment and outcome.

**Conclusions:**

This qualitative study offers new insights into the interprofessional practices of physiotherapists working with adults with low back pain, as perceived by the physiotherapists’ themselves. Based on the results, the development of strategies aiming to increase interprofessionalism in the management of low back pain would most likely require taking into consideration factors associated with patients, providers, the organizations within which they work, and the wider systems.

## Background

In the last few decades, there have been increasing demands for health providers to take part in interprofessional practices and collaboration in health systems everywhere [1-4]. One of the underlying rationales for the promotion of collaboration and interprofessional practices is that such practices improve the healthcare response for adults with complex health problems [5]. Low back pain is one such problem, and a major public health issue with enormous consequences at the individual and societal levels [6,7]. For example, based on the results of a recent systematic review [8], the mean one year prevalence of low back pain is 38.0 ± 19.4% (standard deviation), while associated direct (e.g. medical bills) and indirect (e.g. financial compensation) costs of low back pain add up to several billions of dollars yearly in countries like Canada and the US.

Many clinical practice guidelines recommend offering multidisciplinary or interdisciplinary interventions to adults with low back pain [9-11]. The results of previous studies on the effectiveness of such interventions indeed support the involvement of different providers in the management of this condition [12-14]. Positive outcomes of multidisciplinary or interdisciplinary interventions have been documented for variables such as reported pain, disability, return to work and quality of life [12,14-16].

Physiotherapists are among the providers who regularly contribute to the treatment of adults with low back pain [17]. Faced with people seeking treatment for this health problem, do physiotherapists work alone or do they have interactions with other providers? If they do have interactions, how do these interactions take place? The literature offers little evidence on such questions pertaining to the interprofessional practices of physiotherapists [18]. No study was identified on the interprofessional practices of this group of providers in the context of their interventions with adults with low back pain [19]. Furthermore, although a large proportion of physiotherapy interventions for this population are carried out in the private sector in Canada and elsewhere, interprofessional practices in this sector are understudied, as previous work in rehabilitation has mostly examined interprofessional practices in hospitals or rehabilitation centres [20]. Still, over 42% of physiotherapists worked in the private sector in Canada in 2011 [21].

The complexity of low back pain, the importance of low back pain as a public health problem, and the paucity of research on the interprofessional practices of physiotherapists supported the development of this study. It was anticipated that gaining the physiotherapists’ perspectives and experiences on this subject would help inform physiotherapy practice in the private sector with adults with low back pain. Hence, the objectives of this study were to: 1) explore how physiotherapists who work in the private sector with adults with low back pain describe their interprofessional practices, 2) identify factors influencing their interprofessional practices, and 3) identify effects of interprofessional practices, as perceived by physiotherapists.

## Methods

### Study design

This was a descriptive qualitative study [22], the first part of a larger mixed-methods study that aimed at drawing the portrait of the interprofessional practices of physiotherapists working in the private sector with adults with low back pain. The present study was followed up by a quantitative survey.

### Study frame of reference

In this study, we explored *interprofessional practices* to translate our interest in exploring all forms of interactions between physiotherapists and other providers (e.g. not just team-based interactions), from the same and other disciplines, as well as from within the same and other work organizations.

**Figure 1:**
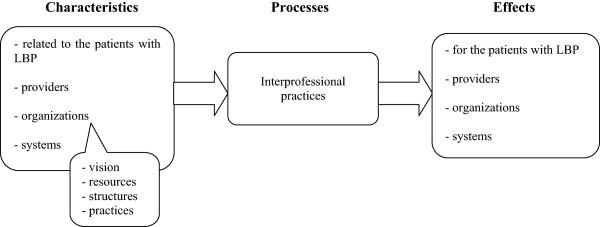
Conceptual frame of reference of the interprofessional practices of physiotherapists working in the private sector with adults with low back pain (LBP).

According to Sicotte et al.’s [23] analytical framework of interdisciplinary collaboration, the intensity of interdisciplinary collaboration in team-based programs is the combination of 1) *interdisciplinary coordination*, or the ability to link together the activities of different providers in time and space (defined by San Martin Rodriguez [24], citing Georgopoulos (1975)), and 2) *sharing care-related activities*, namely collecting information, decision-making, intervention, and assessment of results [23]. To our knowledge, no frame of reference has specifically been developed for the interprofessional practices of physiotherapists. We nonetheless chose to base our study on existing general conceptualizations, while attempting to be context-specific. We adapted Donabedian’s [25] structure-process-outcome model based on the object of study, study objectives, methodology and analyses (Figure 1). According to this frame of reference, patient, provider, organizational and system-level characteristics influence the interprofessionnal practices of physiotherapists (viewed as processes), which in turn have effects on patients, providers, organizations and systems. Furthermore, organizational characteristics were defined using a configurational approach [26] according to which models of organizations are characterized by an organization’s vision, resources, structures and practices.

### Selection of participants

Physiotherapists working in the private sector and who offer interventions to adults with low back pain were the target population for this study. We focussed on physiotherapists who work with this clientele based on the premise that interprofessional practices may diverge for different clienteles. To be included in the study, the physiotherapists had to: 1) be a member of the Order of Physiotherapy of Québec (OPPQ), mandatory to practice in Québec, 2) do clinical work in the private sector at least one day/week, 3) have a minimum 20% of clientele consulting for low back pain, 4) mainly provide interventions to adults, 5) speak, read and write in French, and 6) be located within 300 km of Québec City. Physiotherapists off work at the time of the study (e.g. on maternity leave) were excluded. Our sampling frame, made available to us by OPPQ, consisted in the list of physiotherapists who, as of June 2010, worked in the private sector in Québec, and accepted to be contacted for research purposes (as declared in the annual membership renewal), which totaled 957 out of 1529 physiotherapists working in the private sector. Approximately 42% of physiotherapists work in the private sector in Québec [21].

Multiple case sampling [27] was used. The physiotherapists were first selected randomly within every randomly ordered administrative region of the province. This was followed by maximal-variation sampling, a form of non-probabilistic sampling that consists in searching for participants who will most likely present different perspectives on the object of study based on selection criteria that may influence these perspectives [28]. Inspired by previous studies [29-31], three criteria were retained for their probable influence on the perceptions of physiotherapists regarding interprofessional practices: duration of professional experience (<or ≥ 10 years), work location (rural/urban = < or ≥ 10 000 inhabitants), and proximity with providers from other disciplines (working without immediate proximity of providers from other disciplines, many different providers working close to the clinic but in different organization, or many different providers working in same organization). At least 12 physiotherapists, that is, one physiotherapist most likely fitting each of the combinations of criteria [27], were to be recruited and interviewed. It was planned that a greater number of participants would possibly be required, depending on data saturation. Wishing to maximise the distribution of participants across the territory of the Province, one physiotherapist was initially chosen from each administrative region (n = 10), until a potential participant from each region was identified.

### Recruitment procedure

The study coordinator sent a brief personalized introductory letter to potential participants and called them approximately one week later to describe the study and nature of participation, as well as to verify eligibility. An appointment was then made with interested and eligible physiotherapists. At that time, the coordinator provided more information on the study, obtained written informed consent and proceeded with the interviews with the consenting physiotherapists, of whom none were previously known by the interviewer. A 25-dollar gift certificate was offered to participants as a symbolic recognition for their time. The Ethics Committee of the *Institut de réadaptation en déficience physique de Québec* approved the study (project # 2010–190).

### Data collection

Semi-structured face-to-face interviews served as the main method of data collection. An interview guide was constructed based on study objectives, literature review, frame of reference, as well as discussions with research team, experts and potential users. It was pretested with two physiotherapists, in order to refine questions and the interview procedure [32]. The interviews were carried out by the first author and recorded. Intensive note-taking was undertaken quickly after the interviews in order to synthesize their contents and circumstances and to document interviewer thoughts and impressions [33]. A brief questionnaire on socio-demographic information and professional practice was also completed with the participants after the interviews (except for one who returned the questionnaire later).

### Data analyses

The content of the interviews was transcribed verbatim, noting silences, sighs, laughs and sounds [34] and then imported into NVivo 8 (QSR International) to facilitate data organization and analyses [28]. Data analyses were based on content analysis [35]. General first-level codes were initially predetermined according to our frame of reference, and the list of second-level more specific codes evolved based on collected data [32]. This led to the determination of a first tree-like list of codes, that was constructed in an iterative manner, going back and forth between the framework, the transcripts, the list of codes and extracted sections associated with codes; a process that was documented in a coding journal [34,36]. The unit of analysis was the smallest section of text that had meaning when isolated, as recommended by Bazeley [34]. The codes were then regrouped under more general themes, related between them, and compared, as described by Creswell et Plano Clark [28]. In order to validate the list of codes we constructed, as suggested by Miles and Huberman [37], we proceeded with multiple coding. One interview was fully coded by another member of the research team and the coding of two others was also verified. The minimal divergences obtained were discussed and common understanding rapidly obtained.

## Results

### Characteristics of participants and context of interviews

Thirteen physiotherapists were interviewed, while twenty-two physiotherapists were sent an introductory letter (five series of mail-outs). Indeed, upon making the follow-up phone calls, 4 physiotherapists were not working in the same organization, 2 did not fit the eligibility/sampling criteria and 3 were never reached. All physiotherapists with whom appointments were made accepted to participate in the study. Our sample of physiotherapists comprised 10 women and 3 men having between 3 and 21 years of professional experience (Table 1). They worked in one of 10 regions of Québec, most of them full-time (69.2%). French was their mother tongue and main language used at work. The interviews were conducted in a quiet space and lasted between 55 and 95 minutes.

**Table 1:** Selected characteristics of physiotherapists (n = 13)

**Characteristics**		**N (%)**	**Mean (SD)**	**Median**	**Range**
Gender					
Men		3 (23.1)			
Women		10 (76.9)			
Age (years)			35.6 (7.5)	36.5	25.5 - 46.8
Professional experience (years)			11.0 (7.2)	9.7	3.0 - 21.0
Current physiotherapy work					
Full-time (>27 hours/week)		9 (69.2)			
Part-time (≤27 hours/week)		4 (30.8)			
Hours per week worked			32.1 (12.9)	35.0	6.0 - 57.5
Type of affiliation with work setting					
Owner/Co-owner		5 (38.5)			
Employee		8 (61.5)			
Work in another organization					
Yes		2 (15.4)			
No		11 (84.6)			
Professional experience in current setting (years)			5.7 (4.3)	4.0	0.58 - 15.0
Professional experience working with people with low back pain (years)			11.1 (7.4)	9.0	2.0 - 21.0
Percentage of patients with low back pain in daily practice			47.9 (17.4)	50.0	20.0 - 67.5

### Objective 1: Physiotherapists’ descriptions of interprofessional practices

The physiotherapists used multiple terms to refer to their interactions with other providers, including *teamwork*, *interdisciplinarity*, *working together, collaborating, sharing information/experience/knowledge, coordinating actions, sharing common goals, treating together, referring and respecting each other*. For example, one physiotherapist said:

« for me, to collaborate is to…hum to…to call the doctor or to write to the doctor to tell him where the client’s at and what I think » (V1-12)

The terms used varied between physiotherapists, but also within interviews. When prompted to define terms they used, the physiotherapists often responded showing hesitation in their choice of words or simply answered something like “that’s difficult!”.

Physiotherapists identified different means of interacting with other providers: face-to-face, telephone, letter, fax, unplanned and planned face-to-face meetings, shared files, joint evaluations and referral. Many reflected indirect rather than direct interactions with other providers, such as sending a letter to another provider via the patient or sharing information from a common file. The great majority of interactions with other providers were said to be unplanned rather than planned (e.g. formal meeting).

The other healthcare providers with whom the physiotherapists reported interacting worked in the same setting or not, and in the public and private sectors. The physiotherapists mostly mentioned interactions with medical doctors (family doctors and specialists), but also talked about interactions with other physiotherapists, physiotherapy assistants, psychologists, kinesiologists, massage therapists, acupuncturists, osteopaths, chiropractors, and orthotists. When prompted to discuss other providers they had interactions with, a few physiotherapists also mentioned secretaries, because they often represented intermediaries between themselves and other providers. Claim agents of funding agencies were also mentioned as they were occasionally contacted to obtain permission to refer patients to other providers. The patients themselves were reported as intermediaries between the physiotherapists and other providers, e.g. by relaying information or a letter, by acting upon (or not) physiotherapists’ referrals to another provider or by being directly present during interactions with other providers. The physiotherapists conversely identified some providers mentioned above (e.g. MDs, chiropractors, psychologists) and other types of providers with whom they did not have any interactions, mainly unregulated providers under the Code of Professions of Québec, e.g. bone setters, reflexologists, and orthotherapists. One participant stated:

«…we can work with everybody except (…) for bone setters, for sure that doesn’t pass, we can’t do it (…), and we understand why right away, they have no notion; when we explain, they don’t understand anything (…)” (V1-5)

### Objective 2: Influencing factors

Factors influencing the interprofessional practices of physiotherapists were found to relate to the different categories of characteristics identified in our frame of reference, as presented in Table 2. These factors were found to be highly interrelated.

**Table 2:** Factors influencing physiotherapists’ interprofessional practices

**Categories of factors**	**Reported factors**
*Patient-related*	- Patients’ conditions and needs
	- Financial situation
	- Patient request
*Provider-related*	- Provider attitudes
	- Professional language and treatment orientation
	- Competition for clientele
	- Previous interactions
	- Personal knowledge of other providers and their roles
	- Workload and work schedules
*Organization-related*	- Vision of services involving multiple providers
	- Physical proximity of other providers
	- Shared files
	- Rules regarding interactions with other providers
	- Within- and between-organization activities
*System-related*	- Provider shortage
	- Rules of funding agencies
	- Hierarchy between professions and provider culture

### Factors related to the patient

Physiotherapists explained they had interactions with other providers based on the patient’s condition and needs. When the physiotherapists assessed a patient’s condition and felt they were missing information, had attained their limits, or that intervention was beyond their scope of practice, they were more likely to interact with other providers. For example, the physiotherapists often talked about contacting the referring MDs to suggest further investigation or medication, or to obtain information or discuss. The stage of the condition (persistent rather than acute), the course of recovery, the presence of psychosocial factors, pain location (central or peripheral), and compensation status were all seen as factors that were associated with a greater need for interactions with other providers. One physiotherapist mentioned:

“If recovery is good and it’s ‘business as usual’, well then we won’t make a note for the doctor to say ‘ehh… the patient came to see us, everything is going well’”. (V1-3)

This idea was not conveyed by all physiotherapists however. The patient’s financial situation also emerged as a factor that had an impact on the physiotherapists’ interactions with other providers, mainly in regards of referral to other providers. For example, one physiotherapist explained how when she knew that paying for treatment was difficult for a patient, she would refrain from referring to another provider, even if she thought it was relevant, and also acknowledged that she sometimes cut her own interventions to allow for those of other providers. A patient’s request for physiotherapists to interact with another provider (e.g. send a letter) was also seen as an incentive.

### Provider-related factors

Physiotherapists’ and other providers’ attitudes emerged from the physiotherapists’ discourse as greatly influencing the frequency and quality of interactions. An open attitude and confidence in other providers were seen as important facilitators. Sharing common language and treatment orientation was also viewed as such. One physiotherapist explained that she could not interact with chiropractors because they did not share the same language or vision of treatment. Another physiotherapist talked about competition for clientele he felt from other physiotherapists working in other clinics in the same town when he tried contacting them. Hence, he had mostly abandoned interactions because of their limited interest to discuss. Having had previous positive interactions with or personally knowing another provider was viewed as an incentive for further interactions. Conversely, not knowing other providers, especially their professional roles, and feeling that other providers did not know the roles of physiotherapists or did not see them as credible professionals was expressed as a barrier to interactions. As stated by one physiotherapist:

“…my boss hired the acupuncturist so… If he… if he hadn’t hired her, I wouldn’t necessarily have believed in that. (…) I’m less likely to refer my patients there (…). It’s hard to fake that I believe in it to refer…” (V1-13)

Another physiotherapist mentioned:

“the fact that half the doctors in the region don’t know us, or that they simply don’t want to know us, that for sure doesn’t… doesn’t motivate us to call them and to give them our opinion or to ask them their opinion because we kind of get the impression that (sighs) they don’t have time to lose with us” (V1-10)

Most physiotherapists mentioned they had to be cautious when approaching some MDs, for instance by not being too affirmative in making their observations, in order to prevent unpleasant reactions. In another vein, physiotherapists’ and other providers’ workloads and work schedules were said to negatively impact interactions. Many physiotherapists reported having little time to contact other providers, although they took it, and had difficulties reaching some providers, mainly MDs. As an example:

“…my schedule is loaded. So, with (…) patients every half-hour we don’t have a time-slot (…) to make phone calls for example. (…). So we make them more often when we have two free minutes or when a patient doesn’t show up.” (V1-6)

### Organization-related factors

Organizational factors were interrelated and linked with the organizations’ vision, resources, structures and practices, as proposed initially in our frame of reference. In some organizations, there was a clear vision to provide services from multiple providers, e.g. to be able to satisfy patients’ needs, and take part in interprofessional practices, which acted as facilitating factors for interactions. The physical proximity of other providers was viewed as facilitating. For some physiotherapists, being in the same clinic was essential, but for others, being close by (e.g. same building or across the street) was sufficient. As an example, one physiotherapist talked about the importance of working in the same physical space as other providers, which helped to construct professional credibility in the eyes of other providers:

“The other professionals more precisely know what we do when they see us working. Then they know we’re professionals in our own right.” (V1-2)

The organization’s structures were seen as influencing factors through the use of shared files and the mostly implicit but sometimes explicit rules of the organization regarding interactions with other providers (e.g. formal meetings). For instance, many physiotherapists reported that in their organization, letters were to be sent to the MDs after initial evaluation or at the time of the first follow-up visit after referral for physiotherapy. As for organizational practices, their main influence on the interprofessional practices were perceived through the arrangement of training, promotional and social activities between organizations, such as lunchtime conferences, and within-organization parties, which helped to get to know and create links with other providers that facilitated further interactions.

### System-related factors

The physiotherapists discussed factors related to the healthcare, educational and professional systems. The shortage of providers in some regions was identified as a barrier to interprofessional interactions that even had an effect on modifying physiotherapists’ professional roles. One physiotherapist mentioned going to a private gym to plan an exercise program for a patient and one of her colleagues doing worksite visits to analyze ergonomics, tasks she said they wouldn’t do if a kinesiologist and an occupational therapist, respectively, worked in the region. The rules of the provincial funding agencies were said to play a role in determining the types and timing of interactions with other providers, e.g. forms being sent to the MDs based on requests of funding agencies. The physiotherapists also talked about persisting hierarchy between professions, feeling that physiotherapists’ status was viewed as inferior in the professional system, mostly by some MDs. To this effect, every physiotherapist interviewed judged that it was much easier to interact with younger (more often women, remarked a female physiotherapist) than older MDs, hence shedding light on the influence of timing and content of training and probably changing medical culture. One physiotherapist said:

“Physio evolves a lot, and new doctors now have better knowledge of what physio is. I think it’s really the older ones that are more reluctant.” (V1-7)

### Objective 3: Effects of interprofessional practices

The participants reported mostly positive effects of interprofessional practices and very few negative effects, synthesized in Table 3. They mainly highlighted effects for providers and patients.

**Table 3:** Effects of physiotherapists’ interprofessional practices

***Positive effects***	***Negative effects***
Patient-level:	
- Self-management	- Pain management
- Sense of being “taken care of”	- Patient reactions
- Confidence in providers	
- Timing and quality of recovery	
- Timing of interventions	
- Whole-person consideration	
Provider-level:	
- Harmonization of messages	- Provider reactions and interactions
- Stimulation, peer-support, reassurance, pleasure, satisfaction	
- Knowledge acquisition	
Organization-level:	
- Performance	- Referrals (loss and over-referral)
- Referrals	
System-level:	
- Knowledge and credibility of physiotherapy profession	

For the positive effects, the physiotherapists stated that interprofessional interactions positively influenced patients’ self-management, their sense of being “taken care of”, confidence in providers, more rapid and optimal recovery, and offered better timing of interventions (e.g. for referrals, investigations) and consideration for all aspects of the person. As one physiotherapist put it:

“What I say to myself is: ‘the most important, yes, we are a business here, we need clients to live (…) but that’s because we need them to recover also if we want to live ehh… from our popularity (…). For them to have a better recovery, we don’t have a choice, we need to… to all talk to each other’.”(V1-4)

Similarly, one physiotherapist clearly linked good communication between providers in the organization where she worked and the organization’s good performance. Some physiotherapists also mentioned how interactions prevented from giving mixed messages to the patients. In another vein, the physiotherapists spoke about the stimulation, peer-support, reassurance, pleasure and satisfaction they felt from interacting with other providers, within and outside their own workplace. A physiotherapist said:

“collaborating is… is fun (laughs)! (…) It’s fun because we communicate, and we don’t feel alone. It’s hard to take the the… the pain of our clients on our shoulders alone… So… to be able to exchange with another professional that has another vision, it sometimes helps to find other options. And hum… when we can, we even put the client with us.” (V1-4)

Gaining new knowledge through interactions with other providers was highlighted too. Furthermore, the physiotherapists reported mostly receiving positive reactions following interactions with other providers, as noted by increased referral for their services or to their organization. Having interactions was also a way to inform other providers, mainly MDs, of what physiotherapists do and enhance their credibility through positive results of physiotherapy interventions. One of the physiotherapists expressed:

“Well it’s certain that the doctor who sees that me, my client, well he has to recover and that I refer him all over, well he will be more likely to refer to me because the client is important.” (V1-1)

Many physiotherapists spoke more generally about how their interactions helped to improve the status of physiotherapy and its role within their local health system. As for lack of interactions, it was seen as being associated with higher costs for the system.

For the negative effects, some physiotherapists mentioned having unpleasant conversations with other providers, mainly MDs, mostly regarding professional limits, which made them very careful when sharing information and approaching the other providers, as evoked previously, e.g. avoid saying anything that might be interpreted as having a diagnostic message. One physiotherapist/clinic-owner even recalled having lost all physiotherapy referrals from one MD following a dispute with one of the organization’s physiotherapists. Another physiotherapist described negative effects related to over-referral within his own work organization:

“…referring sometimes to people who are incompetent, that’s one thing (…) ehh or to refer all the time. (…) It’s like, if for some people coming into the clinic: “oh for sure you’re gonna go for massage therapy, for sure you’re gonna go for acupuncture, for sure you’re gonna go for osteo”. No. You know, you have to be careful. That, that could become double-edged if it’s, if it’s not well used.” (V1-11)

Referring to providers in other organizations where physiotherapy was also offered was viewed as potentially leading to loss of clientele, a problem one physiotherapist/clinic-owner resolved by gradually including the types of previously referred-to providers within his own organization. Having too many providers involved in a patient’s management was seen by one physiotherapist as a rare negative consequence with people with pain problems, because of the fact that through their interventions, the providers keep the patients centered on the pain. One physiotherapist stated that for patients who don’t tell the same story to all the providers they receive interventions from, the interactions may be seen as an irritant by them, although these interactions allowed to clarify the situation between providers.

## Discussion

In this study, we explored the perceptions and experiences of physiotherapists who work in Québec’s private sector with adults with low back pain on their interprofessional practices. More specifically, we explored the physiotherapists’ descriptions of their interprofessional practices, as well as perceived influencing factors and effects.

Our exploration of physiotherapists’ descriptions showed that, for them, interprofessional practices include a wide array of processes. Indeed, physiotherapists described their practices in multiple ways, which included action-based activities such as working together and referring. As suspected, only a minority of physiotherapists practiced in contexts where there were formal team-based processes. The physiotherapists mostly mentioned interactions that took the form of unplanned information-sharing between providers, as well as referral to other providers. Hence, physiotherapists’ reported interprofessional practices in the private sector did not perfectly match often-found definitions of interprofessional collaboration that encompass intensive and formal types of interactions, usually between formal team members [38]. Our findings also show that interactions between physiotherapists and MDs are at the heart of physiotherapists’ interprofessional practices, as most of the physiotherapists’ reported interactions involved MDs. However, interactions with other providers were also reported and took similar forms (e.g. unplanned discussions).

Exploring physiotherapists’ perceptions of factors that influence their interprofessional practices allowed to highlight interrelated contributions linked with 1) patients, notably the stage and severity of their condition, 2) providers, such as attitudinal, knowledge-based and reachability factors, 3) organizations, including rules and proximity with other providers, and 4) wider systems, such as lack of providers, administrative constraints and hierarchy between professions. Our study is one of the few that specifically explored patient-related factors influencing interprofessional practices. Interprofessionnal practices and collaboration are often justified in the literature by the need to address the complexity of person or population needs and conditions. Still, how this complexity is defined and the association between complexity and interprofessional practices have rarely been the focus of empirical studies. In Sicotte et al.’s [23] study, acute stage and limited severity of the health problems seemed to more often lead to interventions involving only one provider in walk-in programs, in comparison with complex and chronic problems, a result in line with our own findings. The results of our study provide patient-specific elements expressed by the physiotherapists as sources of complexity in patients with low back pain, who were seen by some as more likely requiring the implication of other providers. As for hierarchy between providers, few studies have specifically addressed issues related to the physiotherapist-MD relationship [18,39,40], none of them in the specific context of practice of the current study. In another vein, factors linked to administrative requirements of funding agencies related to reimbursement for physiotherapy and financial constraints of the patients also appear as newly identified context-specific factors influencing the interprofessional practices of physiotherapists working in the private sector. The influence of other provider or organizational-level factors specifically associated with the reality of private sector practice (e.g. tight schedules) was also highlighted.

Numerous positive effects of interprofessional practices were perceived by the physiotherapists. They include gaining new knowledge as a provider, being valued in one’s own role, as well as improving treatment and outcome. Overall, the physiotherapists considered their interactions with other providers as essential, some wanting further interactions, most not wanting to work without them. In one of the few previous studies carried out in the context of physiotherapy practice [18], physiotherapist-reported benefits of interprofessional collaboration included adoption of a holistic vision and improved quality of services. Our study also underlines effects that are specific to the private sector, such as increased referral for physiotherapy.

### Strengths and limitations

This study mainly focussed on interprofessional practices in an understudied population, physiotherapists, working in an understudied context of practice, the private sector. Faced with this lack of research, using a qualitative approach to explore the subject of interprofessional practices was warranted. Although most elements were redundant in the last few interviews leading us to conclude to data saturation, while deepening the analyses, we found that further interviewing may have helped clarify more of the latent meanings of interprofessional practices and further our understanding of the conceptualizations of the phenomenon. Categorizing the physiotherapists according to our recruitment matrix was also imperfect because on a few occasions, what the physiotherapists reported at the time of eligibility verification over the phone was different from what they stated during the interviews, for example because they had forgotten that another type of provider sometimes worked in their clinic. We nonetheless feel we were able to gather a good diversity of perspectives. However, we did not explore the views and experiences of other providers, as well as service users’, which could further help understand the phenomenon.

### Clinical implications

The findings of this study provide hints for improving the organization of physiotherapy services in the private sector and improving interprofessional practices when relevant. Possible avenues in the workplace include creating occasions to interact with other providers, for example by organizing joint training sessions or social activities and taking steps to gain more knowledge on other providers’ roles (e.g. by reading, asking questions). Encouraging the physical proximity of different providers in the same organization appears as a strategy worthy of further consideration and could lead to the identification of preferred models of organizations. On another level, system-wide professional culture changes and provider training related to interprofessional practices should continue to be promoted based on our results. Furthermore, because our findings highlighted multiple sources of influence shaping the interprofessional practices of physiotherapists, it is most likely that it would not be sufficient to simply ask physiotherapists to increase their interactions with other providers in order to increase such practices. Other context-specific organizational, system-level and patient-related factors would also need to be taken into account and acted upon.

## Conclusions

In these times when interprofessional practices and collaboration are greatly promoted in health systems everywhere, our results offer new insights into the interprofessional practices of private sector physiotherapists working with adults with low back pain, as perceived by the physiotherapists themselves. Based on our findings, the development of strategies aiming to increase interprofessionalism in the management of low back pain would most likely require taking into consideration factors associated with patients, providers, the organizations within which they work, and the wider systems. In the future, additional research focussing on the views of other actors, such as other providers, as well as service users, would enhance our comprehension of physiotherapists’ interprofessional practices.

## Competing interests

The authors declare that they have no competing interests.

## Authors’ contributions

KP led study design, data collection, analysis and interpretation and drafted the manuscript. CED significantly contributed to study design, data collection, and data analysis and interpretation. MR participated in study design. DM participated in study design and data analysis. All authors contributed to manuscript preparation and approved the final manuscript.

## Pre-publication history

The pre-publication history for this paper can be accessed here:

http://www.biomedcentral.com/1471-2474/15/160/prepub
